# Supplementary material for: Are carbon emissions trading and green financial instruments synergistic? -Comprehensive quantitative research based on content analysis
Source: PLoS One. 2024 Mar 7;19(3):e0298601. doi: 10.1371/journal.pone.0298601 (PMC10919601; doi:10.1371/journal.pone.0298601)
Supplement: S1 Data — (ZIP) [file pone.0298601.s001.zip › Data/地级市Z_F工作报告.zip!/║╙▒▒/└╚╖╗╩╨/└┤╫╘╣1⁄2╓┌║┼í╛┬φ┐╦╩2╛▌═°í┐_2013.docx]

各位代表：

　　现在，我代表廊坊市人民政府向大会作工作报告，请予审议，并请各位政协委员和列席人员提出意见。

一、过去五年的工作回顾

　　过去的五年，我们在省委、省政府和中共廊坊市委的坚强领导下，全面贯彻落实科学发展观，遵循“依法、科学、民主、文明”的工作方针，聚焦“京津冀电子信息走廊、环渤海休闲商务中心”发展定位，实施精良管理，追求高端发展，圆满完成了本届政府任期各项目标任务，在实现“两个率先”的征程中迈出了坚实步伐。

　　五年来，我们积极应对经济下行压力，着力增投资强后劲保增长，全市经济进入总量快速扩张、质量效益同步提升的新阶段。综合经济实力显著增强，2012年地区生产总值达到1793.8亿元，年均增长11.3%。全部财政收入突破300亿元大关，从2007年的95.6亿元增加到306.6亿元，地方公共财政预算收入从45.4亿元增加到172.1亿元，分别增长2.2倍和2.8倍，全部财政收入占地区生产总值的比重从10.8%提高到17.1%，各县（市、区）财政收入都已迈过或接近10亿元台阶。规模以上工业增加值达到673亿元，比2007年增长1.1倍。全社会固定资产投资由686亿元增加到1314亿元，实施亿元以上项目1950项，争列省重点项目550项。五年中，全市经济总量由全省第七位上升到第六位，地方公共财政预算收入由第六位跃居第四位，贷款余额由第五位提高到第三位，实现了从“小廊坊”到实力廊坊的历史性跨越。

　　五年来，我们坚定不移推进产业结构调整，实施高端发展和创新引领战略，转变经济发展方式取得明显进展。三次产业结构进一步优化，由2007年的13.2:56.6:30.2调整到2012年的11:54:35。金字塔型现代产业体系拉开框架。总部经济、网络经济、创新经济方兴未艾，华为中国片区总部落户廊坊，润泽国际信息港、中国联通华北基地、汉能全球研发中心等一批高端项目加快建设。先进制造业、现代服务业发展势头强劲，富士康、中轻造纸装备、南玻玻璃、航天振邦、燕达国际健康城、中商国际农产品交易中心、亚太国际家居材料城等一批重大项目建成投产，精雕数控机床、首钢大型冶金装备制造基地、碳纤维研发生产中心、鲁能生态城、爱晚香河国家养老示范基地等项目加快建设。电子信息产业势头强劲，主营业务收入增长3.7倍，高端装备制造业和现代服务业增加值年均分别增长19.3%和16.7%。传统产业转型升级步伐加快，累计完成技改投资1697.4亿元，实施技改项目3527个，新奥集团、汇福粮油、梅花集团等一批骨干企业转型升级。园区承载力不断增强。新获批省级园区30个，廊坊开发区升格为国家级开发区，燕郊高新区成为国家级高新区。科技创新能力进一步提高。共取得科技成果554项，其中409项达到国际国内领先水平，建成国家级重点实验室和技术中心3家，成为国家新型工业化电子信息产业示范基地。节能减排成效明显。以落实“双三十”工程为重点，实施505项减排工程，万元地区生产总值能耗累计下降15.8%，二氧化硫、化学需氧量等主要污染物排放量分别削减19.8%和21.8%，圆满完成省下达任务。

　　五年来，我们坚持统筹城乡一体化发展，壮大新产业、培育新农民、建设新民居，走出了一条适合自身区位和发展阶段的农村发展新路子。农业产业化水平进一步提高，按照城郊都市型、生态集约型定位，引进建设了恒都美业、达利食品等一批龙头项目，农民专业合作社发展到1010家，国家级、省级龙头企业分别达到5家和39家，农业产业化经营率由61%提升到66%，居全省第二位。各项惠农政策全面落实，累计发放各种惠农补贴资金27.9亿元。粮食生产连年丰收，肉蛋奶菜等主要农产品产量稳定增长。新民居和“幸福乡村”建设稳步推进，高标准建设531个省级新民居示范村，建设基础设施和公共服务项目750项，累计培训农民101万人次。

　　五年来，我们以高端规划引领城镇建设，打造区域性高端休闲商务政务平台，城镇格局、功能、品质和管理水平不断提升。城镇规划水平迈上更高层次。按照“生态、智能、休闲、商务”城市定位，编制完成城市总体规划纲要、中心城市空间发展规划等系列规划，全市域“一体两翼”城镇总格局初步确立，中心城市由“小三点、大三点”向“小四点、大四点”组团结构拓展，全市城镇化率达到51%，比2007年提高5.7个百分点。城镇功能显著提高。借势城镇面貌三年大变样，坚持“缓在新区摊大饼、先下笨功改老城”，对周各庄等城中村和棚户区进行拆迁改造，城中村改造完成80%，拆临拆违146万平方米，占既有建筑面积的24%。以“一轴一廊两环八大中心”建设为骨架，打造高水平、现代化的城市核心区和功能性中心，五年累计投入带有公益性的城建资金775亿元，万达广场、京沪高铁站、市体育场建成投入使用，苏宁广场、香港新世界广场、浙商广场等大型城市综合体组团式落户，瑞龙园、丹凤园、自然公园西扩等大型城市园林项目加快建设，梦廊坊文化产业园奠基开工，千年古刹隆福寺重修工程进展顺利。开发区次中心加速构建，会展中心及周边区域功能提升和景观改造工程全面开工。城南面貌发生巨大变化，龙河次中心全面开发建设，创业大厦建成投入使用，红星美凯龙大型城市综合体即将开业运营。县城和重点镇建设水平普遍提升。三河大力实施精品工程，文化中心广场建成开放，汇福国际健康中心、万亩森林公园等大型城市项目加快实施。霸州打造国际化生活品质城市，中国戏曲大观园、温泉公园、牤牛河历史文化公园等建成投入使用。香河打造生态宜居家园，运河大道、城市休闲广场等重点市政工程竣工投入使用。固安建设京南现代田园城市，城镇面貌和功能大为改观，已成为承接北京产业转移的重要基地，各县（市）城镇建设都迈上了新台阶。城镇管理水平进一步提升。城市网格化管理覆盖面继续扩展，在全省率先实施了市、县两级数字化城市管理新模式。

　　五年来，我们着眼破解当前和长远发展制约，着力推进基础设施建设，经济发展的承载力、保障力大幅提升。对接京津、贯通市域的重大交通设施取得历史性突破。全市交通基础设施累计投资217亿元，廊沧高速建成通车，弥补了廊坊南北主路无高速的重大缺憾，大广高速、密涿支线高速全线通车，京台高速加快建设。京沪高铁高密度经停廊坊，使廊坊主城区到北京的时空距离缩短到20分钟以内。全市新改建农村公路2119公里，新增公路通车里程1095公里，路网密度从每百平方公里120公里增加到148公里，居全省第一。北京新机场选址京廊交界，三河通航机场起降点已获批准，将为廊坊打开通往全国乃至世界的更多窗口。统筹推进土地管理，五年争取规划规模、用地指标和挂钩指标三项工作保持全省领先。水资源短缺制约开始有效破解。引黄入廊实现常态化，连续三年累计引水2.2亿立方米，有效缓解了文安、大城两县水资源短缺问题。南水北调配套工程建设进展顺利，廊涿干渠全线贯通，广阳水库建设获批立项，天津干渠廊坊段建设如期竣工，我市中部和南部地区将形成较为完备的供水网络体系。关系未来发展的能源、资源性项目加快建设。国电廊坊大热电项目获批，中电投燃气电站项目加快推进前期工作，大城煤田综合勘探开发进入实质性阶段。五年累计植树造林100万亩，全市森林覆盖率达到24.5%，市区人均公共绿地13.1平方米，在全省率先实施PM2.5监测，荣获中国人居环境奖，成为全国第一个被重新授予国家环保模范城市称号的设区市，三河、霸州跻身首批省级环保模范城。

　　五年来，我们深入推进重点领域改革，加快对外开放步伐，经济发展的动力和活力不断增强。重点领域改革实现新突破。明珠集团、廊雪面粉等9家国有企业完成改制重组，梅花生物科技、华夏幸福基业、廊坊发展等7家企业成功上市。引进河北银行、光大银行等22家银行保险证券机构，组建51家小额贷款公司、5家村镇银行，农村信用社改制进展顺利，实现乡镇银行服务全覆盖。基层医药卫生体制综合改革和县级公立医院改革试点工作全面完成，市区公交实现公车公营，事业单位人事制度改革、农村综合改革有序推进，我市被评为全国文化体制改革先进市。对接京津不断深化。与京津签署一系列合作框架协议，签订合作项目1553项，在规划对接、交通互联、园区共建等方面取得新突破，主城区开通首都机场客运专线，9个县（市、区）引入北京城市公交。招商引资取得新成绩。“5·18”经洽会升格为国家级展会，“9·26”农交会向全国性综合展会迈进，五年实际利用外资25.8亿美元，年均增长12.2%，外资企业发展到737家，其中世界500强企业28家。民营经济快速发展。累计新增注册民营企业1.2万家，增加值、上缴税金分别增长2倍和4倍，5家民营企业进入全国500强，千人拥有民营企业数量始终保持全省领先。

　　五年来，我们在抓好经济建设的同时，始终高度重视推动社会建设同步共进，各项社会事业实现统筹协调发展。科技支撑引领作用得到增强，连续五次荣获全国科技进步先进市称号，成为国家可持续发展实验区。义务教育发展日趋均衡，标准化学校建设全面实施，建成燕京职业技术学院，我市被国务院授予全国“两基”工作先进单位。公共文化服务体系进一步健全，全面完成乡镇综合文化站建设任务，农家书屋实现行政村全覆盖。弘扬“大气、锐气、和气”的廊坊精神，打造龙凤文化品牌，荣获中国龙凤文化之乡和中国书法城称号，霸州成为全国县级公共文化服务体系建设先进典型。基层医疗卫生服务体系不断完善，在全省率先完成农村卫生“三基两化”建设任务，乡镇卫生院全部实行基本药物制度，村卫生室建设任务全部完成，新型农村合作医疗参合率达到96%，城市社区卫生服务街道覆盖率达到100%。

　　全民健身运动广泛开展，竞技体育水平不断提升，圆满承办省第十三届运动会。国防动员和双拥共建深入实施，连续五年被评为“全国双拥模范城”。我市荣获全国人口计生综合改革示范市称号。大厂县获得国家民族地区科学发展示范县荣誉。安全生产主要指标全省领先，社会管理综合治理、信访工作得到加强，社会和谐稳定局面进一步巩固。外事侨务、广播电视、妇女儿童、扶残助残、防震减灾、物价、档案、人防、气象等各项事业取得新成绩。

　　五年来，我们把改善民生作为全部工作的出发点和落脚点，着力做好个人、家庭和市场做不了、做不好的民之所需，民生工作走在全国同等发展水平地区前列。城镇居民人均可支配收入达到25766元，农民人均纯收入10447元，分别居全省第一位和第二位。公教人员收入水平不断提高，津贴补贴标准由全省第四档上升为第一档。城乡就业规模持续扩大，城镇新增就业岗位22.4万个，农村劳动力转移就业116.7万人，城镇登记失业率控制在2%以内，始终保持全省最低。教育资助体系不断完善，基本实现适龄学生不因家庭经济困难而失学。社会保障水平明显提高，城乡居民社会养老保险在全省率先实现制度和地域全覆盖；城镇职工大病报销封顶线由20万元提高到25万元，工伤和失业保险实现市级统筹；连续8次提高企业退休人员基本养老金，城镇低保标准由月人均175元提高到352元，农村由83元提高到152元，保障标准全省领先；率先在全省建立低保家庭临时困难救助制度和支出型贫困家庭救助制度，五保集中供养率全省领先，孤儿救助工作进入全国前列。新建116座联村集中供水水厂，解决了农村156.5万人饮水安全问题。住房保障工作扎实推进，累计建设保障性安居住房48678套，15146户家庭受益，保障对象从低收入家庭扩大到中低收入住房困难家庭，在全省率先实现应保家庭全覆盖。

　　五年来，我们积极转变政府职能，深入推进依法行政和效能革命，政府自身建设取得新成效。坚持科学民主依法决策，重大决策严格履行社会公示、专家论证、风险评估、合法性审查等程序，认真听取民主党派、人民团体和社会各界意见，政府施政更加依法、规范、有序。自觉接受人大法律监督、政协民主监督和社会舆论监督，承办人大代表建议和政协提案2287件，办复率100%。严格规范执法程序和行为，积极推进绩效管理和“效能廊坊”建设，市级行政审批事项减少至100项，五年削减73%。市县全部建成行政服务中心和公共资源交易中心。创新行政管理方式，设立电子台帐，建成项目中心，实现对重点工作和重大项目的动态、远程、实时监管。认真执行党风廉政建设责任制，广泛开展民主评议政风行风活动，强化审计监督，严厉查处各类违纪违法案件，政府的公信力和执行力进一步提高。

　　刚刚过去的2012年，我们积极应对复杂局面、战胜各种困难挑战，全市地区生产总值同比增长9.7%，全部财政收入增长22%，其中地方公共财政预算收入增长22.7%，规模以上工业增加值增长14.1%。着力促升级，制定出台了《关于加快重点产业发展的指导意见》和电子信息、装备制造、现代服务、城郊都市型农业发展实施方案，建设了云存储产业园、北京有研、首钢机电等一批高端项目。着力筑平台，大力推进园区模式创新、机制完善和功能强化，廊坊开发区启动扩区，固安大清河经济开发区与中关村签约共建，空港新区进入实质推进阶段。着力强支撑，协调推进城乡规划建设，谋划实施了城建“双十工程”，36.7平方公里的生态文化艺术新区整体启动，市民服务中心项目顺利获批并开工建设。着力惠民生，全市民生领域投入财政资金208亿元，以前所未有的力度实施就业、住房、医疗、教育、饮水等十项民生工程，解决了农村51.1万人饮水安全问题，超额完成省下达的保障房建设任务，城区实现智能公交全覆盖。面对“7·21”重大汛情，我们紧急动员、全力应对，在全市降雨突破历史极值的情况下，实现了全区域无人员死亡、无责任事故、无重大资产损失。面对十八大维稳安保的重大考验，我们狠抓隐患排查、矛盾化解和源头治理，以超常的工作举措圆满实现了“六个零”的目标。

　　各位代表，回顾过去的五年，我们共同应对了经济下行压力等诸多挑战，承担了北京奥运会、中华人民共和国成立60周年维稳安保等重大任务，及时把握了北京东扩南拓、首都经济圈规划等难得机遇，办成了一批人民群众期待的好事实事，走出了一条适应时代要求、符合廊坊实际的发展道路。五年的发展成就，是市委总揽全局、正确领导的结果，是历届政府励精图治、接续奋斗的结果，是人大、政协和社会各界大力支持，全市人民砥砺奋进的结果。在此，我谨代表市人民政府，向全市人民，向人大代表、政协委员、各民主党派、人民团体及社会各界人士，向驻廊解放军、武警官兵、政法干警和中省直驻廊单位，向所有关心支持廊坊发展的朋友们，致以崇高的敬意和衷心的感谢！

　　回顾过去的五年，我们也清醒地认识到，全市经济社会发展和政府工作中，还存在许多需要着力解决的问题。我市总体实力仍有待进一步提高，新兴产业多处于成长阶段，产业聚集度不高，产业链条不完整，自主创新能力不足，园区档次、功能有待提升，城乡基础设施和公共服务能力还存在较大差距，关系人民群众切身利益的收入分配、就业社保、教育医疗等工作还有待加强。在政府自身建设方面，一些部门服务意识不强、办事效率不高，有些干部还存在小进即满、工作懈怠等问题。对此，我们将采取有力措施，认真加以解决。

　　五年的成就振奋人心，五年的探索弥足珍贵。我们深刻体会到：做好政府工作，必须坚持高端发展不动摇，紧紧围绕主题主线，突出精良管理，追求高端发展，努力实现速度、质量、效益相统一；必须坚持率先发展不动摇，立足区位优势，科学把握市情，深入对接京津、融入京津，着力改善“两个环境”，努力实现“两个率先”奋斗目标；必须坚持创新发展不动摇，以深化改革、扩大开放为动力，大力推进体制创新、管理创新和科技创新，不断为经济社会发展注入新的生机和活力；必须坚持统筹发展不动摇，协调推进工业化、信息化、城镇化和农业现代化，不断推动经济社会协调并进、城乡一体化发展；必须坚持和谐发展不动摇，把维护人民群众利益放在第一位，不断加强社会建设和公共管理，着力保障和改善民生，让发展成果更多更公平地惠及全市人民。

　　各位代表，回首过去是为了更好地开创未来。我们一定倍加珍惜业已形成的基础优势，倍加重视前进中的问题与矛盾，不为困难所阻，不为风险所惧，不为干扰所惑，团结拼搏，开拓创新，勇于担当，不辱使命，在推进“两个率先”的征程中，奋力夺取新的更大的胜利！

二、今后五年的基本思路和主要任务

　　今后五年，是深入贯彻落实党的十八大精神、全面建成小康社会的决定性阶段，也是深入实施“十二五”规划、全面实现“两个率先”的关键时期。廊坊发展具备很多有利条件和积极因素。京津冀区域一体化加速推进，首都经济圈规划正在编制，廊坊将成为北京东扩南拓的直指之所，成为高端要素的汇聚之地。南水北调、引黄入廊、高速路网等重大基础设施加快推进，北京新机场选址京廊交界并获批建设，空港新区加快推进，为廊坊跨越发展创造了重大机遇。我们要深刻认识和全面把握这些有利条件，毫不放松地抢抓机遇，坚定不移地接续奋斗。同时，我们也要对困难和挑战保持清醒的头脑。从宏观形势看，世界经济形势严峻复杂，不确定因素进一步增多。全国各地竞相发展、周边地区增势强劲、省内各市你追我赶，我们面临的压力比以往任何时候都更为紧迫、更为突出。从我们自身看，按照全面建成小康社会的要求，我市实现全省率先的目标仍有不少难点，还需付出艰苦努力。我们必须进一步增强忧患意识、责任意识，以更加积极的精神状态、更加扎实的工作作风、更加坚定的信心决心，迎难而上、抢占先机，努力开创经济社会发展新局面。

　　今后五年，政府工作的基本思路是：坚持以邓小平理论、“三个代表”重要思想和科学发展观为指导，全面贯彻落实党的十八大、省委八届三次全会、市五次党代会和市委五届三次全会精神，更加注重以人为本、更加注重发展质量、更加注重内生型发展、更加注重改革创新、更加注重统筹协调，抓住机遇、乘势而上，保持经济持续健康发展和社会和谐稳定，努力在全省率先全面建成小康社会！

　　今后五年的奋斗目标是：全市地区生产总值年均增长10%以上，全部财政收入、地方公共财政预算收入年均增长13%以上，规模以上工业增加值年均增长13%以上，城镇居民人均可支配收入和农民人均纯收入年均增长10%，经济综合实力显著提升，产业转型升级显著加快，环境建设显著加强，文化事业发展显著进步，人民生活水平显著提高。

　　今后五年，我们将全力推进经济结构战略性调整。围绕“京津冀电子信息走廊、环渤海休闲商务中心”发展定位，深入实施“一产抓特色、二产抓提升、三产抓拓展”战略。大力发展城郊都市型农业，做大做强蔬菜、畜牧、林果花木等特色优势产业，构建优质高效的现代农业发展模式。深入实施工业强市战略，加快培育电子信息、高端装备制造等战略性新兴产业，推动传统优势产业转型升级，积极稳妥淘汰落后产能，推进实体经济扩量提质。加快现代物流、金融保险、会展旅游等现代服务业发展，推动总部、网络、创新三大经济实现重点突破，集中力量打造具有规模效益和市场前景的文化产业聚集区和文化产业项目。电子信息产业增加值占工业增加值比重提高到16%，第三产业增加值占地区生产总值的比重达到50%，产业竞争力明显增强，经济转型取得重大进展。

　　今后五年，我们将全力推进“四化”同步协调发展。坚持把“四化”同步作为推进现代化的基本战略，促进“四化”深度融合。加快新型工业化步伐，努力实现工业经济规模和质量效益全面提升。加快新型城镇化步伐，以产业聚集和人口聚集为核心促进产城融合发展，全面提升城镇功能和规划管理水平，增强城镇综合承载力。加快信息化步伐，推进智慧城市建设，推动信息产业和制造业、服务业融合发展。加快农业现代化步伐，协调推动现代农业和新农村建设，有序推进农业转移人口市民化。全市城镇化率达到60%，城乡基本公共服务均等化程度显著提高，初步形成以工促农、以城带乡、工农互惠、城乡一体、共同繁荣的发展新格局。

　　今后五年，我们将全力推进创新型经济加速发展。坚持把科技创新作为未来发展的战略支撑，着眼发挥自身优势，聚集创新要素、拓展创新平台、构建创新体系，大力推动科技创新、品牌创新、管理创新，以创新驱动引领高端发展。力争研发投入占地区生产总值的比重达到2.5%，新增50家市级以上企业技术研发中心，高新技术产业增加值翻两番，占地区生产总值的比重达到20%以上。空港新区、燕郊高新区、廊坊科技谷、清华科技园等各类创新平台的催化作用充分释放，“廊坊创造”形成品牌群体，高端人才实现近悦远来，推动创新经济成为廊坊的最大特色。

　　今后五年，我们将全力推进改革开放向纵深迈进。坚持把改革开放作为推动发展的根本动力，努力破除制约发展的体制机制等深层次问题，深化行政体制、金融创新、财税体制、国有企业和农村集体产权制度等重点领域改革，基本完成农村土地承包经营权确权颁证，有效释放发展活力和动力。毫不动摇地鼓励、支持、引导非公有制经济发展，深入推进全民创业，推动民营经济上规模、上水平。拓展对内对外开放的广度和深度，利用外资总体规模和效益显著提高。京廊同城发展开创新阶段，努力使廊坊成为京津冀一体化发展的前沿阵地、京津产业转移的首选之地、北京城市转型拓展的新空间。

　　今后五年，我们将全力推进“两个环境”建设。坚持把环境建设作为推进“两个率先”的助推器，以绩效管理和“效能廊坊”建设为抓手，打造更富支撑力和竞争力的发展环境。大力推进南水北调、京台高速、密涿高速主线、唐廊高速、津保铁路等重大基础设施建设，全面增强可持续发展能力。以创建生态市为抓手，以城市水绿生态体系和城乡植树造绿工程为核心，以绿色经济和循环经济为支撑，不断优化生态环境。五年新增造林面积50万亩，森林覆盖率达到29.5%以上，万元地区生产总值能耗、空气质量等生态指标保持全省前列，切实巩固、不断拓展廊坊发展环境、生态环境在全省的优势地位。

　　今后五年，我们将全力推进以民生为重点的社会建设。坚持把改善民生作为政府工作的根本所在，按照保基本、广覆盖、多层次、可持续的方针，加强城乡社会保障体系建设，扎实做好增收、就业、住房、教育、医疗、社保等各项民生工作，加快建立源头治理、动态管理、应急处置相结合的社会管理机制，建立健全基本公共服务体系，统筹推进各项社会事业。根据我市农民收入情况，倒排100个低收入村，开展对口帮扶、定点扶贫。力争城乡居民收入比2010年实现翻一番，衣食住行条件大为改善，全民受教育程度明显提高，劳动就业更加充分，社会保障全民覆盖，文化事业全面繁荣，龙凤文化特色更加靓丽，各项民生社会工作走在全国同等发展地区前列。

　　各位代表，我们肩负着“两个率先”的历史使命，承载着全市人民的信任与期待。面对大潮涌起、风云变幻的发展形势，只要我们坚定攻坚克难的决心，抱定率先发展的信念，就一定能把美好蓝图变成实实在在的发展成果，向全市人民交上一份满意的答卷！

三、2013年目标任务和重点工作

　　2013年是新一届政府的届首之年。做好今年的工作，必须紧紧围绕主题主线，坚持稳中求进的工作总基调，集中力量办大事，聚精会神抓落实，心无旁骛谋发展，万无一失保稳定。

　　全市经济社会发展的主要预期目标是：地区生产总值增长10%左右，全部财政收入和地方公共财政预算收入均增长13%以上，全社会固定资产投资增长20%以上，规模以上工业增加值增长13%以上。城镇居民人均可支配收入和农民人均纯收入均增长10%以上。单位生产总值能耗下降3.66%，污染减排完成省下达任务。

　　围绕上述目标，集中精力做好八项重点工作：

　　（一）强力推进产业结构优化升级。坚持创新型引领、链条式集聚、集群化发展方向，加快构建富有竞争力的金字塔型现代产业体系。一是做大做强重点产业。深入落实四大产业实施方案，大力发展实体经济，着力培育主导产业。电子信息产业，围绕智能终端、平板显示、软件服务等重点领域，加大项目引进和建设力度，加快建设润泽国际信息港、人保集团北方信息中心等一批重大项目。装备制造业，重点培育数控机床、工程机械、航空航天、专用设备、汽车及零部件等支柱产业，加快建设中石油压缩机组维修中心、航天科技产业园、中轻造纸装备二期、香河佳龙消防设备产业园等一批重点项目，打造高端装备制造产业集群。现代服务业，重点抓好汇福国际健康中心、鲁能生态城、香江全球家居CBD、华北油田地下储气库等项目建设，加快壮大现代物流、金融服务、文化创意、休闲旅游产业的规模和实力。城郊都市型农业，以服务都市生活需求为导向，以建设首批现代农业试点县为重点，加快推进三河汇福农产品加工园区、大厂现代农业科技示范园区、霸州达利食品生产基地等项目建设，大力发展休闲农业和农庄型、基地型、订单型农业。二是培育壮大新兴产业。以总部经济、网络经济和创新经济为重点，引龙头、强配套、促聚集，加速推进高端产业集群化、规模化发展。加快国家电子政务外网灾备中心等龙头项目建设，以政府云、金融云、电子商务云等为重点打造云服务基地，促进三河通航机场、华为总部基地、富力总部信息港、安邦保险后援服务中心等总部项目聚集发展，充分发挥新奥能源生态城、汉能全球研发中心等创新经济项目的带动作用。围绕培育壮大新能源、新材料、生物医药等新兴产业，加快建设中广核新奥燃气分布式能源站、洁能国际太阳能、霸州LNG等龙头项目，支持有关县（市）建设生物医药产业基地。三是加速传统产业转型升级。全市完成技改投资490亿元，滚动实施100个省级技改项目，支持企业提高装备水平、优化生产流程、淘汰落后工艺技术和设备，加快金属压延、食品加工、家具制造、包装印刷、保温建材等传统产业升级步伐。四是整合提升园区平台。按照体系化、规范化、高端化要求，大力推进各类园区提质升级。围绕市场前景好、发展潜力大的龙头企业，以及管道、电梯、航天科技等优势资源，建设一批特色产业园区。全力推进空港新区建设，把空港新区建设作为廊坊未来新的战略平台和发展动力，加强与北京方面的沟通协调，积极争取省里支持，高起点编制新区总规和产业、交通规划，力求在征地拆迁、核心区启动等方面取得实质性进展。继续推进廊坊开发区扩区，加快燕郊高新区、廊坊新兴产业示范区、固安大清河经济开发区等重点园区建设。每个县（市、区）集中力量建设一个硬件设施完备、综合功能完善的精品园区，三年内主营业务收入达到300亿元以上，打造县域经济增长极。

　　（二）强力推进重大项目攻坚。坚持把增投资、上项目作为稳增长、调结构的重要支撑，全力实施600个亿元以上项目，继续保持项目建设的强劲势头。在项目谋划引进上求突破。以重点产业实施方案为路线图，加强项目谋划，着力引进一批电子信息、装备制造、现代物流、生物医药、现代农业、新材料和新能源产业项目，优先引进总部经济、网络经济和创新经济项目，力促战略性新兴产业壮大升级。做好现有项目的挖潜延伸，围绕增资扩产、延长产业链条，招商储备一批优质项目，使项目建设始终保持开工、在建、储备连续推进的高效状态。在争列省重点项目上求突破。密切跟踪省重点项目建设政策，加大争列推进力度，力争省重点项目数量进入全省第一方阵。借助全省百家央企、百家高校院所、百家知名民企进河北活动，力争签约落地一批重大产业支撑项目。同时筛选确定100项市管重点项目，各县（市、区）分别确定一批重点项目。在项目建设上求突破。大力度、高水平、快节奏推动项目建设，重点推进建设一批投资10亿元以上项目。加快中电投燃气电站、维特根亚太生产基地、大城煤田综合开发等签约项目开工建设，加快国家已核准的国电廊坊大热电等5个项目和美国JM新型管材、中国联通华北基地等在建项目建设进度，推动中建机械制造基地、世原汽车配件三期等已投产项目尽快达产。在项目服务管理上求突破。千方百计破解土地、资金制约，为项目建设提供优质高效服务。坚持市级领导分包重大项目责任制，紧盯项目进度，严控项目质量，解决项目难题，全程做优项目服务。采取项目拉练和分片调度相结合的办法，一季一调度、一月一通报，加强项目督导，加快建设进度。全面落实《廊坊市项目建设考核试行办法》，科学考核，兑现奖惩，使之成为衡量项目工作优劣的新标尺。

　　（三）强力推进新型城镇化进程。坚持走集约、智能、绿色、低碳的新型城镇化道路，实施“中心扩容、两翼并驱，服务带动、工业支撑，分区统筹、区域联动”发展战略。一是提升城市规划。借助北京新机场建设，充分发挥京沪高铁的拉动作用，统筹规划城市空间、功能节点和重点产业布局。积极推进城市总规修编及报批，完善市域综合交通、生态保护、市政基础设施等支撑体系规划，完成万庄新城战略规划编制。积极指导各县（市、区）编制完善县城规划和城乡总体规划，统筹做好全市域和县域间规划衔接及产业、交通、城镇等规划的统筹衔接。二是优化城镇体系。以空港新区为突破口，实施中心城市扩容，推动城市组团发展，加快主城区、固安县城、永清县城、万庄新城、廊坊开发区、廊坊新兴产业示范区同城化进程。以县城为支撑，以重点镇、园区为节点，推进以业兴城、以城促产、产城互动，大力推进城镇建设管理，提升城市经济，促进产业壮大和人口聚集，加快培育环北京城镇群和环天津城镇群。三是建设重点工程。推进主城区城建“十项重点工程”，高标准规划建设生态文化艺术新区和中央商务区，加快建设市民服务中心、梦廊坊文化产业园、光明商务岛、城市交通中心等重大工程。全面实施道路提升项目，推进新奥艺术大道东连西延、永兴路南延等道路工程。做好北京新机场建设及周边路网前期工作。推进城北污水处理厂、新水源地建设，实施雨污管网改造和热电联产配套管网建设。县城和重点镇继续实施“五个一”工程，制定完善县城及重点镇建设管理考核标准，促使集聚功能、风貌特色、管理服务上水平。四是提高城市管理水平。开展“城市管理提升年”活动，推进城市管理体制改革，加强规划执法监管，实施环境综合整治，大力推进城区净化、绿化、亮化、美化、序化工程，营造现代、时尚、精致、靓丽的城市环境。完善城市数字规划，拓展数字城管监管范围，将供水、供电、供气、供热服务监管系统纳入城市管控系统和网格化管理，建立地下管线设施数据库，提升城市管理效率和水平。

　　（四）强力推进县域经济发展。把县域经济作为“四化”同步的结合点，统筹推进城乡发展，力促产业升级、财税增长、后劲增强、城乡共进。一是增强县域综合实力。实施县域经济腾飞计划，以项目建设为抓手，着力引进一批高技术、高效益、低能耗的龙头型、基地型项目，促进县域实力增强，五年实现全市县域经济总量倍增。鼓励强县增创优势、率先发展，扶持弱县深挖潜力、跨越发展。全市县级财政收入两年内全部迈上10亿元台阶，三年内多数县（市、区）迈上30亿元台阶，三河市突破百亿。二是壮大县域特色产业。充分发挥各地优势，以县域特色产业为重点，谋划建设中小企业园区，引导特色产业进园区、建基地，打造竞争力强、特色鲜明、配套完善的产业集群。加快传统产业技改步伐，每个县（市、区）实施2～3项区域支柱产业提升工程。大力发展劳动密集型产业、服务业和小微企业，扩大就业面，带动农民群体致富。三是发展现代农业。围绕创建国家现代农业示范区，大力推广“园区+龙头企业+农民合作社+农户”四位一体的现代农业经营模式，以打造现代农业综合服务平台、建立支农贷款担保体系和发展农民专业合作社为支撑，加快构建新型农业经营体系，重点实施100项农业产业化大项目，培育10个农业产业化示范园。推动农村土地承包经营权有序流转，引导工商资本进入适宜产业经营的种养业，发展多种形式的适度规模经营，提升农业产业化经营水平。四是建设“幸福乡村”。启动建设100个省级幸福乡村和新民居示范村，推进南北两个新农村示范片区和8个新农村精品示范点建设，推动美丽乡村、宜居乡村等六村联创。统筹推进城乡规划、基础设施、产业布局、公共服务协调发展。加强低收入村街对口帮扶，新改建农村公路100公里，建设8项110千伏及以上输变电项目，推动新民居碳纤维采暖示范项目，全面提升城乡公共服务、基础设施均等化水平。

　　（五）强力推进改革开放。把改革开放作为破解难题、增强动力和活力的根本途径，全面提升改革开放的深度和广度。一是深化重点领域改革。进一步推进国企改革，引导支持企业上市融资，加快德基机械等企业上市步伐，年内新增3家上市企业。深化财政体制改革，做好“营改增”改革准备工作，推进预算绩效管理，提高资金使用效益。加快村镇银行组建和农村信用社改制工作进程，推动建立京津冀金融一体化综合改革试验区。推进供销社综合改革，深化医药卫生体制和经营性国有资产监管体制改革。二是扩大对内对外开放。做大招商平台，精心组织好“5·18”经洽会、“9·26”农交会等重大会展活动。大力发展会展业，制定促进会展业发展意见，围绕主导产业组织专业会展，培育引进中国国际管道展、中国（廊坊）汽车用品交易会等大型展会。提高招商实效，以产业规划和实施方案为“路线图”，精心谋划一批有影响、有实效的招商推介活动，大力开展产业链招商、专业招商、小团组招商，引进一批大项目、好项目，实际利用外资6.82亿美元。三是深入对接京津。充分利用首都经济圈规划编制和北京新机场建设的机遇，加强与国家部委和北京的沟通协调，力争规划中更多更好地体现廊坊的利益诉求，特别是在空港新区规划建设上，加强深入对接，赢得更大支持。积极吸引京津高科技、高成长企业落户廊坊，全面加强与央企、京企、津企的战略合作，努力在园区共建、资源共享、产业互补等方面实现突破。四是推动民营经济快速发展。全面落实促进民营经济发展的政策措施，完善公共服务平台，推动全民创业，支持引导个体工商户向中小微企业转变，加强民营企业家队伍建设，培育壮大规模以上企业群体。新改建35家中小企业创业辅导基地，围绕科技信息、法律维权、市场营销、信用评价，强化创新创业服务。力争主营收入10亿元以上企业达到50家。

　　（六）强力推进创新驱动。坚持把科技创新作为高端发展的战略支撑，制定发展创新型经济和加快科技创新的实施意见，强化政策引导，提高科技创新能力，全市规模以上高新技术产业增加值同比增长30%以上。一是提升创新平台。着力构建集聚创新资源、汇聚创新资本、凝聚创新人才的高端平台，用好中省驻廊科研机构、高等院校和科研人才，推动新奥研究院、中石油勘探开发研究院等创新平台做大做强，支持龙河高新技术产业区、固安工业区争列省级高新区，推进煤基低碳能源、河北省矿井灾害防治2家重点实验室建设。二是增强创新能力。进一步提升现有研发中心和孵化器创新能力，有效发挥廊坊科技谷、清华科技园等创新平台作用，建设创新经济发展的增长极；强化企业的创新主体地位，新建5～10家市级以上企业技术研发中心、3～4家科技企业孵化器；着力实施科技专项，着眼推动电子信息、装备制造、清洁能源等重点产业升级，开展100项技术攻关；培育自主知识产权和自主品牌，申请专利2000件，新增国家级驰名商标和名牌产品5个；促进科技成果转化，组织百家重点企业、百家京津院所、百家融资机构开展合作对接，推进政产学研紧密结合。三是强化政策落实。切实执行国家和省各项创新政策、人才政策及我市高层次创新创业人才政策，全力引进培养各类专业技术人才、技术领军人才；开展科银企对接服务专项行动，破解科技创新的资金瓶颈，加强政策宣传和政策引导，努力营造有利于创新人才成长的良好环境。

　　（七）强力推进环境建设。把环境改善融入到经济社会发展各领域，贯穿到改革发展稳定全过程，体现到生产生活各方面，实现廊坊环境的大提升。努力改善发展环境。围绕建设“效能廊坊”，扎实推进绩效管理和行政执法规范化建设，提高电子监察系统网络对接、同城审批效率。推行政府部门服务标准化建设，以窗口单位和服务行业为重点，制定服务标准，创新服务方式。深入开展行风评议活动，严厉整治“慵、懒、散、慢”和不作为、乱作为行为，努力实现低商务成本、低生活成本、低行政成本、高品质服务。提升金融生态环境，积极引进域外金融机构，推进金融机构县域全覆盖，加大金融支持实体经济力度，积极防范金融风险。鼓励中介服务机构加快发展，逐步完善以劳动就业、技能培训、法律服务、科技评估为重点的中介服务体系。全面构建信用奖惩和联动体系，形成覆盖全社会的社会信用综合管理框架。不断提升基础设施环境，加快京台高速、津保铁路建设进度，推进密涿高速主线、唐廊高速前期工作。争取采留线升级105国道，实施一批干线公路养护改造工程。加快建设保沧干渠和广阳水库，实施廊涿干渠应急供水，争取更多引黄调水指标，缓解水资源短缺状况。大力优化生态环境。以创建生态市为统揽，主城区构建“一廊、两环”水绿生态体系，重点实施龙河水系综合治理、自然公园西扩和12公里绿道绿廊建设；县域高标准推进生态走廊、植树造绿、农业综合开发等系列工程，启动永定河流域综合治理，建设森林防护生态带，全市完成植树造林20万亩。全面加强环境保护，严格环境监管和环保执法，加强市区PM2.5监测治理，三河、霸州启动国家环保模范城创建，促进环境质量持续提升。深入推进节能减排，严格控制能耗，实施100项重点治污减排工程。不断优化资源利用，继续实施最严格的耕地保护制度、节约用地制度和水资源管理制度，积极发展循环经济和节能环保产业，推广应用清洁能源，加快形成绿色发展长效机制。

　　（八）强力推进民生改善。按照统筹兼顾、突出重点、尽力而为的原则，重点实施10项民生工程。(1)扩大就业工程。健全城乡公共就业服务体系，重点做好城镇就业困难人员、高校毕业生就业和农民培训、农村劳动力转移工作，城镇新增就业岗位4.6万个，完成农民培训20万人次，农村劳动力向非农产业转移5.7万人，城镇登记失业率控制在2.5%以内。(2)教育提升工程。市一中迁建开工建设，力争三年建成。启动市十六中新建、7所中小学改扩建工程。创建130所标准化学校，全面完成校舍安全工程建设任务。启动农村小学附设幼儿园试点，规范发展民办幼儿园，缓解入园难入园贵问题。(3)卫生强基工程。市中心医院开工建设，三年建成投入使用，努力建成全市龙头、全省领先、地级一流的现代化医院。整合建设市卫生应急指挥中心，增加急救站点，充实人员设备，提高急救处置能力。县级实施7所医院、5个急救站、8个卫生监督所、19个乡镇卫生院和3个社区卫生服务中心建设。加强医学高端人才培养和基层卫生技术人员培训，提高医疗卫生人员执业能力和水平。(4)社保提升工程。继续推进社保扩面提标，进一步提高城乡居民医保覆盖面，新农合参合率达到95%以上；城乡居民养老保险参保率保持在90%以上，市区居家养老服务站建设覆盖80%社区；建立覆盖市县的新农合、城镇医保、医疗救助“三网合一”机制；完善社会救助制度，启动城乡低保信息“阳光工程”，城乡低保和五保供养标准在全省继续领先。(5)住房保障工程。加大保障性住房建设力度，全年建设1.7万套。强化分配环节的透明度、规范度，完善后续管理工作，加强公共配套设施建设。推进城区旧小区和棚户区改造，实施蔡豆庄二期、八大街南外街旧改。(6)饮水安全工程。投资2.55亿元建设22座水厂，解决51.1万人饮水安全问题，提前全部实现农村饮水安全目标。(7)文体惠民工程。加快文化艺术中心、市群艺馆建设，推动各县（市、区）“两馆一站”提升改造，在全市谋划建设一批特色博物馆、收藏馆。加快体育公园、市区健身绿道等工程建设，进一步完善全民健身服务体系。(8)环境洁净工程。开展治脏、治乱、治癣集中行动，建设精品街区和样板街区，加强城中村和城乡结合部环境卫生管理。以“四清”为重点，推进农村环境综合整治，改善农村生活环境。(9)城市便民工程。围绕解决好群众身边的生活难题，继续实施“十小便民工程”，改造30条小街巷、30个无物业小区，提升20个小游园，新建一批智能便民服务岛、停车场、农贸市场、物品回收市场、便民警务亭等服务设施。(10)乡村商贸工程。推进万村千乡市场工程，农家店覆盖100%的乡镇和85%的行政村，改扩建3个农产品批发市场，加快推进鲜活农产品流通创新，减少流通环节，降低流通成本。

　　加快发展各项社会事业。继续推进国家可持续发展实验区建设，构建创新创业综合服务平台。促进城乡教育协调发展，进一步加强教师队伍建设，大力推进标准化学校建设，积极发展高等教育和职业教育。统筹推进公共文化事业发展，积极申报第二批国家级公共文化服务体系建设示范区，打造龙凤文化品牌，创作推出一批凸显廊坊文化特色的精品力作。加强全民健身服务，健身设施覆盖80%社区和40%村街，全力备战省第十四届运动会，高水平举办国际热气球节，推动群众体育和竞技体育协调发展。提高公共卫生服务均等化水平，县级公立医院全部推行补偿机制、支付改革和管理机制综合改革，行政村卫生室全部实施基本药物零差率销售，构建以区域卫生信息平台为核心的智慧医疗和数字卫生体系，提升医疗卫生服务水平。建立覆盖全市的食品药品安全风险监测网络，深入开展隐患排查和专项整治，严厉打击食品药品违法犯罪行为。加强全民国防教育，增强国防观念，深入做好拥军工作和国防后备力量建设，争创“全国双拥模范城”六连冠。切实加强法律服务。促进民族团结进步事业发展，支持大厂争创全国民族地区综合改革试验区。加强人防、气象、物价、审计、人口计生、妇女儿童、防灾减灾等社会建设，不断促进各项事业繁荣发展。

　　全力维护社会和谐稳定。加强和创新社会管理，深入开展社会矛盾和信访稳定大排查、大调处活动，切实提高突发事件应对处置能力。提升城乡社区服务管理水平，推进社工、社会组织和社区建设协调发展。切实强化安全生产，突出抓好消防和道路交通安全，坚决遏制重特大事故发生。加强流动人口管理服务。全面推进“京畿平安示范区”建设，营造安定和谐的社会环境。

　　各位代表，我们已经踏上为全面建成小康社会而奋斗的新征程，做好开局之年的工作意义重大、影响深远。我们一定不遗余力抓发展，尽心竭力惠民生，全力以赴促和谐，让廊坊人民生活得更加舒适、更加舒心、更加幸福！

四、加强政府自身建设

　　面对新的形势和任务，各级政府必须切实转变政府职能，大力加强自身建设，努力建设服务政府、法治政府、创新政府、效能政府和廉洁政府，不断提高推动科学发展的能力和水平。

　　第一，坚持解放思想，提高创新发展、高端发展的能力。把改革创新精神融入政府工作全过程，克服固步自封、小进即满的保守惰性思维，以高端发展为目标，以精良管理为标尺，创新发展理念、创新工作方法、创新体制机制，努力在推动高端发展、实现率先发展上探索新路子、实现新突破，使各项工作更加体现科学性、增强针对性、富于创新性。

　　第二，坚持总揽全局，提高统筹兼顾、协调各方的能力。准确把握政府工作的系统性和复杂性，不断提高统筹各方、协调推进的本领，统筹改革发展稳定全局，统筹城乡、县域和经济社会发展，统筹各方面利益关系，充分调动各方面积极性，努力形成全市上下协调联动、合力攻坚、竞相发展的崭新局面。

　　第三，坚持转变职能，提高依法行政、科学决策的能力。着力加强经济调节、市场监管、社会管理、公共服务，切实提高为经济社会发展服务、为人民服务的能力和水平。进一步强化依法行政意识，严格依照法律和程序行使权力，自觉接受人大及其常委会的法律监督、工作监督，自觉接受人民政协的民主监督和社会监督，重视发挥新闻媒体和社会公众的监督作用，充分听取各方面意见建议，做到科学决策、民主决策、依法决策。

　　第四，坚持改进作风，提高凝神聚力、推动落实的能力。认真落实中央“八项规定”，进一步改进工作作风，从市政府领导班子做起，要求别人做到的，自己先要做到，要求别人不做的，自己坚决不做。大兴学习之风，坚持学习工作化，工作学习化，增强工作本领，不断提高解决实际问题的能力。大兴实干之风，坚持深入实际、深入基层调查研究，坚持讲实话、干实事，言必信、行必果，牢记空谈误国、实干兴邦，夙夜在公，勤勉工作，以政府的“辛苦指数”，提升城市的“发展指数”和群众的“幸福指数”。

　　第五，坚持反腐倡廉，提高从严治政、廉洁从政的能力。反腐倡廉必须常抓不懈，拒腐防变必须警钟长鸣。坚持标本兼治、综合治理、惩防并举、注重预防，大力推进惩治和预防腐败体系建设，切实做到用制度管权、管人、管事，确保权力规范运行、透明运行、阳光运行。扩大审计监督范围，严控“三公”支出，厉行勤俭节约，杜绝奢侈浪费，建设节约型机关，从严治政、廉洁持政，以良好业绩造福于民，以良好政风取信于民。

　　各位代表，宏伟的事业激励着我们，人民的期待鞭策着我们，美好的前景鼓舞着我们。让我们在省委、省政府和中共廊坊市委的坚强领导下，咬定青山不放松、面对困难不退缩、团结拼搏不懈怠，加快推进“两个率先”进程，为创造无愧于历史、无愧于时代、无愧于人民的辉煌业绩而努力奋斗！
